# Predictors of visit frequency for patients using ongoing chiropractic care for chronic low back and chronic neck pain; analysis of observational data

**DOI:** 10.1186/s12891-020-03330-1

**Published:** 2020-05-13

**Authors:** Patricia M. Herman, Sarah E. Edgington, Eric L. Hurwitz, Ian D. Coulter

**Affiliations:** 1 RAND Corporation, Santa Monica, CA USA; 2 Office of Public Health Studies, University of Hawaii, Honolulu, HI USA

**Keywords:** Chronic low back pain, Chronic neck pain, Chiropractic care, Visit frequency, Behavioral model of health services use

## Abstract

**Background:**

Chronic spinal pain is prevalent, expensive and long-lasting. Several provider-based nonpharmacologic therapies have now been recommended for chronic low-back pain (CLBP) and chronic neck pain (CNP). However, healthcare and coverage policies provide little guidance or evidence regarding the long-term use of this care. To provide one glimpse into the long-term use of nonpharmacologic provider-based care, this study examines the predictors of visit frequency in a large sample of patients with CLBP and CNP using ongoing chiropractic care.

**Methods:**

Observational data were collected from a large national sample of chiropractic patients in the US with non-specific CLBP and CNP. Visit frequency was defined as average number of chiropractic visits per month over the 3-month study period. Potential baseline predictor variables were entered into two sets of multi-level models according to a defined causal theory—in this case, Anderson’s Behavioral Model of Health Services Use.

**Results:**

Our sample included 852 patients with CLBP and 705 with CNP. Visit frequency varied significantly by chiropractor/clinic, so our models controlled for this clustering. Patients with either condition used an average of 2.3 visits per month. In the final models visit frequency increased (0.44 visits per month, *p* = .008) for those with CLBP and some coverage for chiropractic, but coverage had little effect on visits for patients with CNP. Patients with worse function or just starting care also had more visits and those near to ending care had fewer visits. However, visit frequency was also determined by the chiropractor/clinic where treatment was received. Chiropractors who reported seeing more patients per day also had patients with higher visit frequency, and the patients of chiropractors with 20 to 30 years of experience had fewer visits per month. In addition, after controlling for both patient and chiropractor characteristics, the state in which care was received made a difference, likely through state-level policies and regulations.

**Conclusions:**

Chiropractic patients with CLBP and CNP use a range of visit frequencies for their ongoing care. The predictors of these frequencies could be useful for understanding and developing policies for ongoing provider-based care.

## Background

Chronic low back pain (CLBP) and chronic neck pain (CNP) are the most common types of chronic pain, [1, 2] and their estimated combined adult prevalence is between 10 and 20% [1, 3–11]. This pain is associated with substantial co-morbidity, [12] and is expensive to the healthcare system [13] and to employers [14]. Chronic spinal pain is also long-lasting, with average pain durations from years to decades [15–19].

Many patients with CLBP and/or CNP use medications to manage their pain, including opioids [12, 20]. However, due to the dangers of opioid abuse, recent efforts have focused on finding effective nonpharmacologic therapies [21]. Guidelines now recommend a number of nonpharmacologic therapies for CLBP and CNP [22–24]. Several of these therapies (e.g., acupuncture, cognitive-behavioral therapy, multidisciplinary rehabilitation, spinal manipulation) require ongoing visits to providers.

Unfortunately, the ongoing long-term provision of provider-based care for chronic spinal pain, including appropriate visit frequencies, is not well-addressed in health and payer policies [25–27]. To some extent this is because many of these policies are based on a curative model [28, 29]—i.e., X number of treatments and you should be cured. These policies can require documentation of continued clinically meaningful improvement with the implication that treatment ends when a patients’ symptoms plateau at some maximum therapeutic benefit [30–35]. However, although many policies recognize that care beyond this plateau—i.e., chronic pain management [32] or ongoing or support care [35]—might be needed under certain conditions—e.g., if symptoms deteriorate with treatment withdrawal [30–32, 36]—little guidance and no evidence is offered for this care. This lack of information and support of ongoing pain management has been cited as one barrier to the use of recommended provider-based nonpharmacologic therapies for chronic spinal pain [37].

Information on how patients with CLBP and CNP utilize ongoing provider-based nonpharmacologic care may be useful to the development of better policies in support of this care. It is estimated that 30% of patients with spinal pain have visited a chiropractor, one type of recommended provider-based nonpharmacologic care [38]. In particular, it would be useful to examine the treatment frequency patients used, and the determinants of those frequencies. These determinants include patient characteristics but may also include characteristics of the treating chiropractors.

This study took advantage of data gathered from a large national sample of patients with CLBP and CNP who were using chiropractic care [15]. Other analyses of these data have shown that this sample matches the age, gender, racial, ethnic, income and education levels seen in other national samples of chiropractic patients [15, 38–42]. It has also been shown that these patients have been in pain for an average of 14 years and have been using chiropractic care for an average of 11 years [15]. The majority have a goal of pain management (not cure) [43]. A study of their stated willingness-to-pay for pain reduction indicated that what they value is the maintenance of their current, generally mild pain levels, [44] and on average their symptoms over the 3-month study period are mild (average pain intensity of 3 and 4 on a 0–10 scale with minimal-to-moderate back dysfunction [45] and mild neck dysfunction [15, 46]) and may improve slightly [47]. This last may indicate that they have plateaued at maximum therapeutic benefit. However, on average these patients reported that their pain would double if they did not see their chiropractor, [15] which could be an indication of their experience with treatment withdrawals. In this study we examined the range of visit frequencies (visits/month) these patients used to determine the effect on this behavior of baseline insurance coverage, patient characteristics and the characteristics of the treating chiropractor.

## Methods

This study uses observational data collected over 3 months from a national sample of US chiropractors and their patients with CLBP and CNP. These data were gathered between June 2016 and February 2017 as part of a larger project which has been described elsewhere [48, 49]. Data collection and general characteristics of the sample were also documented in detail elsewhere [15, 50–52]. In brief, this study used a 4-level multistage systematic stratified sampling plan (regions/states, metropolitan areas, chiropractic providers/clinics, and patients) to recruit 20 chiropractic clinics and their patients from each of 6 US regions: Dallas, Texas; Minneapolis, Minnesota; Portland, Oregon; San Diego, California; Tampa, Florida; and Seneca Falls/Upstate, New York. Chiropractors were selected to reflect US national proportions of provider gender, years of experience and patient load as shown in the 2015 Practice Analysis Report from the National Board of Chiropractic Examiners [53].

To minimize the burden on clinic operations each clinic front office staff were asked to offer each patient who visited the clinic over the 4-week recruitment period an iPad upon which to fill out a brief prescreening questionnaire. After that point all patient screening, consent and data collection occurred online and did not involve the clinic. Those who filled out the prescreening questionnaire, met those criteria (> 21 years of age; English proficient; no present personal injury/workers compensation litigation; have low back or neck pain) and gave their email address were sent a longer online screening questionnaire to determine their eligibility for the study—i.e., patients with chronic low-back pain and/or chronic neck pain (i.e., pain of > 3-month duration before starting chiropractic care or patient-reported chronicity). Eligible patients gave consent, answered several additional questions and then were sent an online baseline survey followed by five shorter biweekly follow-ups to collect data on ongoing care and symptoms, and a longer endline survey at 3 months. At each step, in addition to the survey link respondents were sent up to 3 email reminders and if they missed a survey they were allowed to rejoin the series at any point past baseline. Patients received online gift cards for their response to each questionnaire and those who completed all nine questionnaires received a total of $200. This study used visit data from all surveys. However, all other data came from the baseline and screening questionnaires. The chiropractor at each clinic also filled out a questionnaire about their own and their clinic’s characteristics. Because the evidence for nonpharmacologic therapies has focused on non-specific CLBP and CNP, in this study we excluded patients from our sample if they reported that a health practitioner told them that their CLBP and/or CNP was caused by one of a list of medical conditions including cancer, rheumatoid arthritis, spinal stenosis, primary fibromyalgia or fracture.

We specified and estimated 2 sets of multi-level models. The first set examined the predictors of chiropractic visit frequency for those with only CLBP or with both CLBP and CNP but who said that their CLBP was worse (hereafter referred to as those with CLBP). The second set examined the predictors of visit frequency for those with only CNP or with both but said that their CNP was worse (hereafter known as those with CNP).

### Measures

Visit frequency was based on patient self-reported recall of chiropractic use over the past two weeks gathered every two weeks for 3 months. Visit frequency was defined as the average number of chiropractic visits per 30-day month. If patients missed data collection points they were asked for their number of visits since their last data collection and numbers were adjusted accordingly. Patients who dropped out or ended care before the end of the first month were excluded from the analysis. The variables we hypothesized to predict visit frequency were all measured at baseline and are listed below in descending order of their hypothesized importance following Anderson’s Behavioral Model of Health Services Use [54–56].

We measured the enabling factors of patients’ baseline insurance coverage and resulting out-of-pocket costs for chiropractic care in three ways and chose the one that best explained visit frequency. The first is a dichotomous variable for having some coverage or not. The second provides more detail on any limitations (e.g., visit or dollar caps) on coverage. And since insurance coverage has a direct effect on the amount the patient pays out-of-pocket per visit, we also considered including these costs instead.

Patients’ need factors included the characteristics of their baseline pain and function. We included their pain 0–10 numerical rating scale or NRS, [57] and the 10-item Neck Disability Index (NDI) [58] score for those with CNP and the 10-item Oswestry Disability Index (ODI) [45] score for those with CLBP. These are all recommended, valid and reliable (pain NRS [59–63]; NDI [46, 64–66]; ODI [67–69]) measures. We also included what patients reported at baseline as to their duration of pain and whether a respondent had both CLBP and CNP. Research has shown that those with both types of chronic pain have worse outcomes, [70] and duration of pain may be a justification for ongoing care [32].

Certain lifestyle or point in care characteristics could also be considered need factors affecting visit frequency. Higher physical demands (i.e., time spent in heavy labor or physically demanding tasks) have been hypothesized as a justification for ongoing care [32]. Also, being a new (< 30-day) patient or a patient who is getting ready to end care may affect visit frequency.

Patients’ goals for their care may reflect perceived need factors and affect visit frequency. These goals were elicited in the baseline survey using an item asking for those with CLBP: Which of the following best describes what you hope to get from your chiropractor regarding your low back pain? The four response options were: Prevent low back pain from coming back or prevent re-injury; Prevent low back pain from getting worse; Ease low back pain or make low back pain go away temporarily; and Make low back pain go away permanently (cure). Those with CNP received a similar item and set of responses but asking about neck pain.

Pain beliefs may also be considered perceived need factors and were captured using several measures including patient report of whether they believed their pain was chronic, and what would happen to their pain on a scale of 0–10 if they did not see their chiropractor. This last question was added after pilot studies indicated that this seemed to motivate patients more than their current pain levels, and later analyses confirmed this focus [44]. Patients also reported their level of agreement (1 = agree to strongly agree) with a series of statements about chronic pain, including that chronic pain will never go away, it is important for me to understand what causes my pain, and it is unsafe for someone with my condition to be physically active (a rough measure of fear avoidance [32, 71, 72]).

Certain psychological variables may also be perceived need factors and associated with visit frequency. Patients’ self-efficacy for their pain management was measured at baseline using that 5-item subscale of the Chronic Pain Self-Efficacy Scale [73] and used patient response averages across items from 1 = very uncertain to 10 = very certain as to their ability to accomplish each statement. Expectations were measured using two items from the Credibility/Expectancy Questionnaire: how successful your chiropractor will be in reducing your pain (1 = very/extremely successful), and how much improvement in pain do you expect over the next 3 months (1 = a lot/quite a bit of improvement) [74]. Expectancy has been shown in other studies to positively effect outcomes [75] and was suggested as one justification for ongoing care [32]. Worry and anxiety are associated with worse outcomes, [72, 76] and may be related to treatment frequency. We included an item regarding how often a statement that I worry all the time about whether pain will end is true (1 = to a moderate or great degree/all the time). Those who are depressed have worse outcomes, [71, 76] and may be justified to receive ongoing care [32]. We used the 4-item depression scale from the PROMIS-29 v2.0 and indicated those with mild depression or above (scores > 52.5) [77, 78]. Finally, there is some evidence that pain catastrophizing is associated with outcomes, [75, 79–81] and may affect patients’ visit frequency. We measured catastrophizing using the sum of 0–4 scores from 3 items asking how often these statements are true: I worry all the time about whether the pain will end, I think the pain is never going to get any better, there is nothing I can do to reduce the intensity of the pain.

Finally, we included several predisposing factors (age, gender and education) to examine their impacts on visit frequency. Several studies have found that those who are older respond less favorably to treatment, [17, 82, 83] and age may be a justification for ongoing care [32]. CLBP outcomes have also been found to be associated with level of (Bachelor’s degree or higher) education [84, 85].

Variables for clinic (chiropractor) and region (state and metropolitan area) were used to determine whether visit frequency was clustered by clinic or region. If clustering by clinic was found, we added chiropractor/clinic characteristics to the model to see if they explain how visit frequency varied by clinic, including average number of patients treated per day, percentage of patients seen for preventive/maintenance care, years in practice, clinic location as urban (> 50,000 population [86, 87]), and whether and how many of five physical modalities (electrical therapy, heat therapy, hydrotherapy, ice therapy, and ultrasound) were used. We also examined the effect of the college where the chiropractor trained. Finally, if clustering by region was found we included state in which the clinic is located to capture any effects of differences in licensure or insurance coverage by state.

### Analysis

We used hierarchical linear modeling (HLM, aka multi-level modeling or mixed models [88–90]) to account for the potential clustering of patients within clinics and regions/states. HLM offered several statistical benefits, including that it corrects for error structure violations (e.g., non-independent errors [88, 91]).

We first ran unconditional (no predictor variables) HLM models to determine whether visit frequency was clustered by region and/or by chiropractor/clinic. We used the Bayesian Information Criterion (BIC) fit statistic (smallest value) to choose the best unconditional model in terms of clustering variable [92]. The BIC was also used to choose the best form of the coverage variable to include in the models. If the difference between BIC statistics for two models is greater than 10, that can be considered “very strong evidence” that the model with the lower BIC is a better fit to the data [93].

Blocks of explanatory variables were then entered into each model sequentially according to a defined causal theory [94]. The work by Andersen and others provided the basis for the models [54–56]. Each block contained a set of related available variables believed to define the causal mechanism in question: enabling, need, perceived need and predisposing factors. The blocks were ordered from those hypothesized to be most proximal to those most distal in explaining an individual’s use of chiropractic care. Because visit frequency could also be affected by chiropractor/clinic characteristics (clustered by chiropractor/clinic) and/or by region even after patient-level characteristics are included, those variables were added last. Sequential entry of these blocks allowed the incremental explanatory power of each block to be measured and tested in a logical order using likelihood-ratio tests. The estimated coefficients reported were derived from the final models (i.e., the models containing all blocks of variables), but only shown for the blocks of variables found to add significant explanatory power (*p* < .05) for at least one condition. The *p*-values for each reported coefficient are provided as an indication of the variables providing the most explanatory power in that block.

Averages and frequencies for the variables considered as potential predictors of patients’ visit frequency were compared between the CLBP and CNP samples using t-tests and χ^2^ tests, respectively. All analyses were performed in Stata 16.0. This study was approved by the RAND Human Subjects Protection Committee.

## Results

**Fig. 1:**
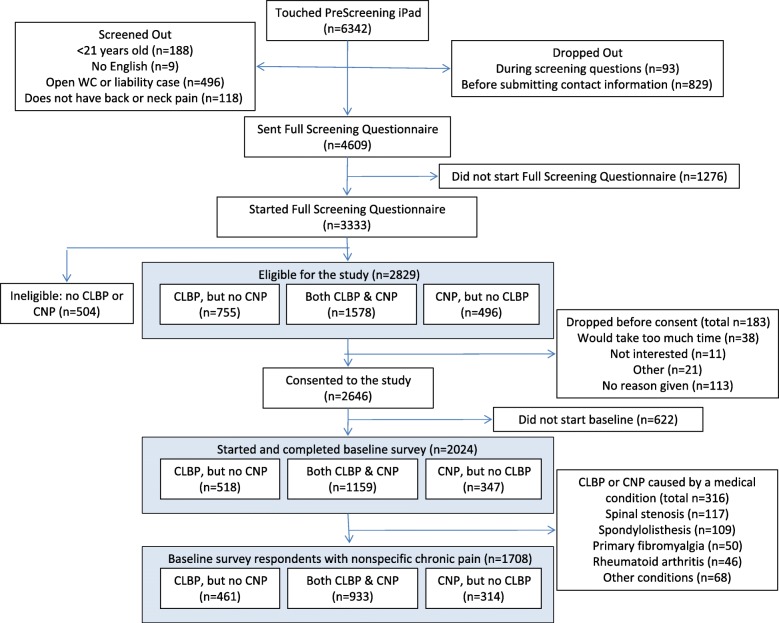
Patient flow into the study. CLBP = Chronic low back pain; CNP = Chronic neck pain; WC = Workers compensation

Of the 2024 chiropractic patients with CLBP and CNP who completed the baseline survey, [15] 1708 met our criterion for non-specific CLBP or CNP (Fig. 1). Of these 1557 (91.2%) patients of 124 chiropractors had sufficient data (including at least one month of data on their chiropractic visits after baseline) to be included in our analyses—852 with non-specific CLBP and 705 with non-specific CNP.

Our unconditional HLM models indicated that the variance in visit frequency across patients was significantly clustered by chiropractor/clinic (*p* < .001 for both the CLBP and CNP models)—i.e., visit frequency was at least partially explained by the chiropractor seen. Clustering by region was not significant and the lowest BIC was found for an unconditional model that only clustered by clinic (Appendix Table A.1). The BICs for the models clustered by clinic were 50 (CLBP) and 44 (CNP) points lower than models with no clustering.

Table 1 shows the averages and frequencies of the variables included in each of the three blocks we considered to represent coverage status for chiropractic care: some coverage/no coverage (columns), more detail on coverage characteristics, and out-of-pocket costs per visit. As can be seen those with some coverage paid significantly less out-of-pocket for care for both conditions (*p* < .001).

**Table 1:** Alternate versions of enabling factors (coverage for chiropractic and costs per visit) to be tested

	Chronic Low Back Pain^1^		Chronic Neck Pain^1^	
	No coverage (*n* = 218)	Some coverage (*n* = 590)	No coverage (*n* = 202)	Some coverage (*n* = 475)
Mean (SD) # chiropractic visits/month	2.0 (1.6)	2.4 (2.4)	2.3 (2.1)	2.2 (2.0)
*Enabling factors*				
Insurance with caps on visits or costs	–	291 (49.3%)	–	244 (51.4%)
Insurance with no caps	–	85 (14.4%)	–	68 (14.3%)
Insurance with unknown caps	–	214 (36.3%)	–	163 (34.3%)
Out-of-pocket costs per visit*				
Unknown / $0	0 (0.0%)	67 (11.4%)	0 (0.0%)	69 (14.5%)
$1 to $20	0 (0.0%)	227 (38.5%)	0 (0.0%)	148 (31.2%)
$21 to $40	14 (6.4%)	195 (33.1%)	19 (9.4%)	175 (36.8%)
$41 to $60	101 (46.3%)	65 (11.0%)	89 (44.1%)	51 (10.7%)
$61 to $80	71 (32.6%)	18 (3.1%)	72 (35.6%)	18 (3.8%)
$81+	32 (14.7%)	18 (3.1%)	22 (10.9%)	14 (2.9%)

^1^Data on insurance status were not available for a small percentage of patients in each sample: 44 (5.2%) for chronic low back pain and 28 (4.0%) for chronic neck pain

^*^Out-of-pocket costs differ significantly between those with and without some chiropractic coverage for both those with chronic low back pain and chronic neck pain, p < .001

Table 2 shows the means and frequencies of each of the predictor variables by block for each sample. The average visit frequency per month was 2.3 for both conditions, a bit more often than every other week. Median visits per month were 1.7 for both conditions and visit frequency ranged from 0 to 14.7 per month for CLBP and 0 to 13.6 for CNP [data not shown]. Almost half have had their pain for 10 or more years and few (7% of those with CLBP and 4% of those with CNP) ended their care (both no visits since last data collection point and report that care has ended) during the last 2 months of the 3-month study period. As can be seen these two samples were remarkably similar. The main exceptions were that CNP patients reported significantly higher pain intensity, worse function, were more likely to have both CNP and CLBP, had somewhat different goals for treatment, were younger, and were more likely to be female. Appendix Table A.2 gives the average visit frequency seen across each of these variables.

**Table 2:** Means and frequencies of each predictor variable in each sample in order of model entry

All figures are n (%) unless otherwise indicated	Chronic Low Back Pain (*n* = 852)	Chronic Neck Pain (*n* = 705)	p-value for difference between groups
Mean (SD) # chiropractic visits/month	2.3 (2.2)	2.3 (2.0)	.900
*Enabling factors*			
Some insurance coverage for chiropractic	590 (69.2%)	475 (67.4%)	.520
Unknown insurance coverage	44 (5.2%)	28 (4.0%)	
*Need factors – pain characteristics*			
Rating of pain past 7 days (0–10), mean (SD)	3.7 (2.1)	4.0 (2.1)	.004
Oswestry or Neck Disability Index score (0–100), mean (SD)	20.5 (12.5)	22.6 (12.5)	.001
Have both CLBP and CNP	444 (52.1%)	428 (60.7%)	.001
Years of pain			.278
Less than 1 year	107 (12.6%)	80 (11.3%)	
1 year to less than 2 years	58 (6.8%)	38 (5.4%)	
2 years to less than 5 years	115 (13.5%)	112 (15.9%)	
5 years to less than 10 years	134 (15.7%)	133 (18.9%)	
10+ years	418 (49.1%)	324 (46.0%)	
Unknown	20 (2.3%)	18 (2.6%)	
*Need factors – lifestyle and point in care*			
No heavy labor	401 (47.1%)	375 (53.2%)	.063
Non-workday heavy labor: > 0% but < 20%	108 (12.7%)	68 (9.6%)	
Workday heavy labor: > 0% but < 20%	163 (19.1%)	140 (19.9%)	
Non-workday heavy labor: > 20%	30 (3.5%)	15 (2.1%)	
Workday heavy labor: > 20%	87 (10.2%)	58 (8.2%)	
Heavy labor: missing	63 (7.4%)	49 (7.0%)	
New patient (< 30 days)	94 (11.0%)	73 (10.4%)	.763
Unknown time with this chiropractor	145 (17.0%)	113 (16.0%)	
Ended care during study period	57 (6.7%)	29 (4.1%)	0.027
*Perceived need factors – goals for treatment*			.001
Goal: prevent pain getting worse	120 (14.1%)	84 (11.9%)	
Goal: prevent pain coming back; prevent reinjury	178 (20.9%)	107 (15.2%)	
Goal: ease or make pain go away temporarily	268 (31.5%)	290 (41.1%)	
Goal: make pain go away permanently	276 (32.4%)	218 (30.9%)	
Goal: other or missing	10 (1.2%)	6 (0.9%)	
*Perceived need factors – pain beliefs*			
Believe their pain is chronic	572 (67.1%)	474 (67.2%)	.967
What pain would be if didn’t see chiropractor 0–10, mean (SD)	6.8 (2.3)	6.9 (2.2)	.179
Chronic pain will never go away: agree/strongly agree	235 (27.6%)	198 (28.1%)	<.001
Important to understand causes of pain: agree/strongly agree	801 (94.0%)	669 (94.9%)	.452
Unsafe to be physically active: agree/strongly agree	53 (6.2%)	25 (3.5%)	.016
*Perceived need factors – other psychological influences*			
Pain management self-efficacy (0–10), mean (SD)	7.5 (1.8)	7.7 (1.7)	.061
Expect chiropractic very-extremely successful	611 (71.7%)	542 (76.9%)	.021
Expect a lot to quite a bit of improvement	538 (63.1%)	447 (63.4%)	.916
Worry about pain: mod to all the time	152 (17.8%)	116 (16.5%)	.471
Has depression according to PROMIS items	215 (25.2%)	188 (26.7%)	.521
Catastrophizing (0–12 scale), mean (SD)	2.3 (2.3)	2.2 (2.3)	.389
*Predisposing factors*			
Age in years	48.5 (14.9)	45.8 (13.2)	<.001
Female	558 (65.5%)	579 (82.1%)	<.001
Education: At least a 4-year degree	462 (54.2%)	413 (58.6%)	.085
*Chiropractor practice characteristics*			
Average number of patients treated per day	25.3 (12.8)	25.6 (12.3)	.612
Percentage of patients on preventive/maintenance	23.7 (17.3)	24.1 (17.4)	.660
In practice 5 to 10 years	100 (11.7%)	84 (11.9%)	.598
In practice > 10 to 20 years	268 (31.5%)	222 (31.5%)	
In practice > 20 to 30 years	258 (30.3%)	231 (32.8%)	
In practice > 30 years	226 (26.5%)	168 (23.8%)	
Clinic location is urban (> 50,000 population)	541 (63.5%)	477 (67.7%)	.086
Average number of physical modalities used	3.3 (1.4)	3.2 (1.4)	.849
*College from which chiropractor graduated*			.344
Life College	79 (9.3%)	63 (8.9%)	
National College of Chiropractic [fix order later]	46 (5.4%)	42 (6.0%)	
Los Angeles College of Chiropractic	28 (3.3%)	29 (4.1%)	
New York Chiropractic College	90 (10.6%)	54 (7.7%)	
Northwestern Chiropractic College	212 (24.9%)	199 (28.2%)	
Palmer Chiropractic College	106 (12.4%)	94 (13.3%)	
Parker Chiropractic College	73 (8.6%)	45 (6.4%)	
Texas Chiropractic College	81 (9.5%)	58 (8.2%)	
University of Western States	106 (12.4%)	97 (13.8%)	
Other	31 (3.6%)	24 (3.4%)	
*State/Region*			.038
California	130 (15.3%)	125 (17.7%)	
Florida	71 (8.3%)	56 (7.9%)	
Minnesota	208 (24.4%)	199 (28.2%)	
New York	155 (18.2%)	99 (14.0%)	
Oregon	117 (13.7%)	111 (15.7%)	
Texas	171 (20.1%)	115 (16.3%)	

We first separately added each of the three options for coverage and out-of-pocket costs to the unconditional HLM models clustered by clinic (Appendix Table A.3). These runs indicated that the best version of the coverage variable block (lowest BICs by 10–12 points for both models) had 3 categories: some coverage, unknown coverage, and no coverage as the reference. We then sequentially added each of the other variable blocks to models with this coverage variable. Table 3 reports in *italics* the explanatory power (statistical significance) of each block when it was first entered. As can be seen the blocks of perceived need factors (treatment goals, pain beliefs, and other psychological variables) were not explanatory when added after enabling or need factors for either model. Estimated coefficients from the final models were reported in Table 3 for each variable in the blocks with significant explanatory power in at least one model. In general, the same coefficients remained statistically significant as additional blocks of explanatory variables were added. All estimated coefficients with their 95% confidence intervals are shown in Appendix Table A.4.

**Table 3:** Hierarchical linear model results predicting chiropractic visit frequency for patients with chronic spinal pain

	Chronic Low Back Pain (n = 852)	p-values for coefficients in block	Chronic Neck Pain (n = 705)	p-values for coefficients in block
*Enabling factors - p-values for block*^1^	*0.032*		*0.986*	
Some insurance coverage for chiropractic – Reference = No coverage	0.44	.008	0.14	.408
Unknown insurance coverage	0.06	.845	−0.15	.689
*Need factors – pain characteristics - p-values for block*^1^	*<.001*		*<.001*	
Rating of pain past 7 days (0–10), mean (SD)	0.06	.238	0.00	.972
Oswestry or Neck Disability Index score (0–100), mean (SD)	0.02	.024	0.03	<.001
Have both CLBP and CNP	0.09	.508	0.15	.309
Years of pain - Reference = < 1 year				
1 year to less than 2 years	0.38	.231	0.36	.307
2 years to less than 5 years	−0.26	.338	− 0.07	.787
5 years to less than 10 years	− 0.29	.259	− 0.11	.674
10+ years	−0.39	.088	−0.03	.894
Unknown	−0.10	.839	−0.01	.986
*Need factors – lifestyle and point in care - p-values for block*^1^	*<.001*		*<.001*	
Heavy labor - Reference = No heavy labor				
Non-workday heavy labor: > 0% but < 20%	0.41	.055	−0.28	.244
Workday heavy labor: > 0% but < 20%	0.47	.010	−0.06	.732
Non-workday heavy labor: > 20%	0.23	.533	−0.02	.971
Workday heavy labor: > 20%	0.22	.350	0.19	.458
Heavy labor: missing	0.43	.104	0.30	.282
New patient (< 30 days)	0.63	.006	0.69	.005
Unknown time with this chiropractor	0.32	.082	0.45	.019
Ended care during study period	−1.21	<.001	−1.57	<.001
*Perceived need factors – goals for treatment - p-values for block*^*1*^	*0.636*		*0.237*	
*Perceived need factors – pain beliefs - p-values for block*^1^	*0.063*		*0.766*	
*Perceived need factors – other psychological factors - p-values for block*^1^	*0.295*		*0.100*	
*Predisposing factors - p-values for block*^1^	*0.048*		*0.171*	
Age in years	0.01	.104	0.01	.111
Female	−0.37	.013	−0.12	.525
Education: At least a 4-year degree	0.04	.805	−0.15	.307
*Chiropractor practice characteristics - p-values for block*^1^	*<.001*		*<.001*	
Average number of patients treated per day	0.03	<.001	0.04	<.001
Percentage of patients on preventive/maintenance	0.01	.259	0.01	.020
Years in practice - Reference = 5 to 10 years				
In practice > 10 to 20 years	−0.50	.072	−0.16	.567
In practice > 20 to 30 years	−0.72	.014	−1.01	.001
In practice > 30 years	0.24	.487	−0.13	.704
Clinic location is urban (> 50,000 population)	−0.20	.318	−0.18	.387
Average number of physical modalities used	−0.12	.067	−0.04	.624
*College from which chiropractor graduated - p-value for block*^1^	*0.130*		*0.060*	
*State/Region - p-value for block*^1^	*0.014*		*0.072*	
State/Region - Reference = California				
Florida	−0.67	.096	−0.66	.109
Minnesota	−0.57	.625	−1.31	.205
New York	−1.49	.001	−1.20	.010
Oregon	0.48	.558	0.20	.802
Texas	−0.30	.574	−0.05	.927

^1^P-value for the likelihood-ratio test of the incremental explanatory power of each block of variables added to a model containing all previous blocks

Having some insurance coverage increases visit frequency by 0.44 visits per month for patients with CLBP but has little to no effect for CNP. Having worse function (higher ODI/NDI scores) and being a newer patient increased visit frequency for both CLBP and CNP. For those with CLBP having some heavy labor at work increased visit frequency and being female decreased visit frequency. On the other hand, those who ended care during the last 2 months of the 3-month study period already had significantly fewer visits before their decision to end.

Even after accounting for all available patient characteristics, the characteristics of the treating chiropractors provided significant explanatory power to patients’ visit frequency. Visit frequency was significantly higher for patients of chiropractors who reported treating more patients per day and was significantly lower for patients of chiropractors who have been in practice for 20–30 years. Visit frequency was also significantly higher for patients with CNP when the chiropractor reported a higher proportion of patients being seen for preventive/maintenance care. The block for the chiropractor’s college of graduation did not add significant explanatory power to either model. However, after controlling for all patient and chiropractor characteristics visit frequency was still significantly lower for patients in New York state. After the last block was added to each model, the variance in visit frequency by chiropractor/clinic was no longer significant meaning that the variables added successfully captured the influence of the chiropractor on patient visit frequency.

## Discussion

In these samples of patients with CLBP and CNP who were using ongoing chiropractic care, average visit frequency was 2.3 chiropractic visits per month—i.e., just over one visit every two weeks. This visit frequency was found to vary significantly by the characteristics of the patients, the characteristics of the treating chiropractors, and the state in which care was given.

Patients with CLBP and some insurance coverage for chiropractic had on average less than one-half visit more per month than those without coverage. However, insurance coverage had little to no effect on visit frequency for patients with CNP. In other analyses about two-thirds of this sample said that having insurance coverage was very or extremely important to their use of chiropractic care [15]. However, those with coverage still face a variety of barriers to care that could limit visit frequency. These include high out-of-pocket expenses and other (e.g., travel, missed work) costs for every visit, visit limits, and prior authorization requirements [26]. Also, because we gathered these data from June of one year into February of the next, generally over the last months of a plan benefit year, we may have captured use of chiropractic for some patients after their coverage for the year had ended due to caps or limits.

As would be expected, patients with either CLBP or CNP and worse function had significantly higher visit frequency. Either going from the mid-point of minimal to the mid-point of moderate disability on the ODI for patients with CLBP (an increase of 30 points [45]) or going from the mid-point of mild to the mid-point of moderate disability on the NDI for patients with CNP (an increase of 20 points [46]) increased visit frequency by an average of about two-thirds of a visit per month. Patients’ pain levels were not explanatory after controlling for coverage and other pain characteristics. It is also not surprising that those who were new patients (< 30 days with this chiropractor) had about two-thirds of a visit more per month, and those who were about to end care had significantly lower visit frequency—roughly monthly on average. Also, for patients with CLBP having some heavy labor increased visit frequency by less than half a visit per month and female patients had lower visit frequencies by just over a third of a visit per month. It is unclear why women with CLBP have fewer chiropractic visits per month than men.

Including all available patient characteristics explained some of the variation of visit frequency seen by chiropractor—i.e., some chiropractors tend to see more of certain types of patients. Nevertheless, including all available patient characteristics did not fully explain the variation in visit frequency seen by treating chiropractor. The characteristics of the chiropractor/clinic also had an influence on visit frequency. Patients of chiropractors who report treating more patients per day, and for CNP who report a higher proportion of patients receiving preventive/maintenance care, have significantly higher visit frequencies, and patients of chiropractors who have been in practice for 20–30 years have significantly lower visit frequencies. The patients of a chiropractor who treated double the average number of patients per day (50 rather than 25) had on average three-quarters to one more visit per month, and patients of chiropractors who have been in practice 20 to 30 years have roughly one less visit per month than those in practice for 5 to 10 years.

There has been concern amongst payers and providers that patients’ use of ongoing care was not based on medical necessity or need, but on clinician dependence or coercion, including through lowered patient self-efficacy or heightened patient fear [32, 95–97]. We did find that a patients’ visit frequency was to some extent determined by the chiropractor from which they received treatment. However, patient self-efficacy or worry did not seem to make a difference in visit frequency. In fact, the pain management self-efficacy scores in this sample were fairly high, and in line with patients’ general belief that they have control over their pain [43]. Chiropractors who treat more patients per day also seem to have their patients return more often. This could simply be a strategy to generate more revenue, or there could be a valid relationship between shorter visits and the need for more visits. We also found that the patients of chiropractors who had been in practice 20 to 30 years had fewer visits than those of chiropractors who had practiced for 5 to 10 years. This effect could either be associated with an established practice’s lower need to generate revenue, and/or that treatment from a more experienced practitioner reduced patients’ need to return as often. The effects of a chiropractor’s treatment approach and experience on visit frequency could be worthy targets for subsequent research. In any case, average patient visit frequency did not vary greatly across the chiropractors in our sample. Only 2 out of our sample of 124 chiropractors had average patient visit frequencies that were more than 2 standard deviations above the overall average of 2.3 visits per month—i.e., more than 6 visits per month [data not shown].

Finally, even after controlling for patient and chiropractor characteristics there seems to be some effect on visit frequency of being treated in New York State. Visit frequencies for patients with CLBP or CNP being treated in New York were more than one visit per month lower than patients being treated in California. This may be because of stricter coverage rules for chiropractic care in New York State.

Other studies have reported on visit frequency for chiropractic care. One study compared practice patterns over a year for patients with CLBP in chiropractic (*n* = 51) and primary medical care (*n* = 14) clinics [98]. They found an average number of visits per year of 6.7 for chiropractic care (median = 4; range = 1–56); just over an average of half a visit per month. The same numbers for medical care were 1.9 visits per year (median = 1; range = 1–17). Another study examined the use of chiropractic services from 1985 through 1991 in the US and Canada [40]. Across US sites the average duration of an episode of care for low back pain was 61.7 days and involved an average of 15.6 visits, so an average of 7.8 visits per month during an episode of care. It is possible that our estimate of an average of 2.3 visits per month is between these two previous estimates because we only followed our patients for 3 months (more could have ended or reduced their visit frequency over a year) and we didn’t require patients be in an episode of care (no more than 30 days elapsed between visits) for the entire 3-month period.

### Limitations

This study benefits from extensive data collected from a large sample of patients using chiropractic for chronic non-specific spinal pain and from their chiropractors. Nevertheless, our study has limitations; the main one being that the sample used in this study is not a representative sample of all patients with CLBP and CNP. It is, however, a reasonably representative sample of chiropractic patients with CLBP and CNP [15] in terms of age, [38–42] having at least partial insurance coverage for chiropractic, [39, 41, 42] and high prevalence of women, [39–42] non-Hispanic whites, [39, 41, 42] those with higher income [42] and more education [39, 42]. The sample is also representative in terms of pain and function. Since everyone in our sample was receiving ongoing chiropractic care, we would expect that their pain and function levels would lie somewhere between the baseline and end-of-study scores seen in trials. For example, compare the baseline and end pain NRS (0–10) and ODI scores seen in a trial of manipulation for CLBP (baseline: pain = 6.0, ODI = 29.5; end-of-study: pain = 3.0, ODI = 13.7) [19] to our sample’s scores of 3.7 and 20.5. Also, compare the baseline and end pain and NDI scores seen in a trial of manipulation for CNP (baseline: pain = 5.6, ODI = 27.9; end-of-study: pain = 3.5, NDI = 19.5) [18] to our sample’s scores of 4.0 and 22.6. Although similar gender, racial/ethnic, income, and education profiles have also been found for those who used other types of nonpharmacologic therapy for back and neck problems, [38] our study’s results should not be generalized to patients who are not now using these therapies. Nevertheless, this sample provides a real-world snapshot of how these patients were using chiropractic care over time.

Given the concern that patients utilize ongoing chiropractic care due to reasons such as clinician dependence or coercion for provider financial gain, another limitation of our study is that it would have been helpful to have a measure of whether patients’ responses were based on what they were told by their chiropractor versus their lived experience. Their having lived with their pain condition for an average of 14 years and the consistency of their responses across the sample [15] give weight to responses based on lived experience. However, the variation of visit frequency by chiropractor also indicates their influence. Our analyses are also subject to the usual limitations of self-reported data. For example, we used patient report rather than administrative claims data to determine visit frequency. Whereas, claims data can be more accurate than patient recall, claims data would have only been available for those with insurance coverage and would not include the patient and chiropractor characteristics used in this study. However, despite these limitations, our models were able to account for all the variance in patient visit frequency attributable to the chiropractor/clinic. Note that this study did not examine the outcomes experienced by these patients, which would be important for the development of effective policies. Outcomes for this sample are addressed in the next paper in this series.

## Conclusions

According to *NIH Medline Plus*, a publication of the National Institutes of Health, “chronic pain usually cannot be cured, but it can be managed.” [99] Several provider-based nonpharmacologic therapies have been recommended for chronic spinal pain, and these therapies may be used on an long-term ongoing basis by patients for pain management. Despite this need, ongoing provider-based care is not well-addressed in the evidence or supported in health and payer policies, [25–27] and this adds another barrier to the use of these recommended nonpharmacologic therapies [37]. This study examined data from a large sample of patients with CLBP and/or CNP to see how these real-world patients used chiropractic care over time to manage their pain. Our sample patients’ high pain management self-efficacy and long-term experience living with their conditions make them good source for information on how ongoing provider-based care for pain management might work. Chiropractic patients with CLBP and CNP manage their pain using a range of visit frequencies and the predictors of these frequencies could be useful for developing policies for ongoing provider-based care.

## Supplementary information

**Additional file 1: Appendix Table A.1.** Results of the unconditional models; **Appendix Table A.2.** Average visit frequency for each category of each predictor variable in each sample; **Appendix Table A.3.** Models testing the options for insurance coverage and its effect on out-of-pocket visit costs; **Appendix Table A.4.** Full results of the final hierarchical linear models predicting chiropractic visit frequency for samples of patients with chronic low back pain and chronic neck pain.

## Data Availability

The datasets generated and/or analysed during the current study are not publicly available due to this provision not being included in participants’ consent forms but are available from the corresponding author on reasonable request.

## Abbreviations

BIC: Bayesian Information Criterion
CLBP: Chronic low back pain
CNP: Chronic neck pain
HLM: Hierarchical linear modeling
NDI: Neck Disability Index
NRS: Numerical rating scale
ODI: Oswestry Disability Index
