# Supplementary material for: Predictors of visit frequency for patients using ongoing chiropractic care for chronic low back and chronic neck pain; analysis of observational data
Source: BMC Musculoskelet Disord. 2020 May 13;21:298. doi: 10.1186/s12891-020-03330-1 (PMC7222295; doi:10.1186/s12891-020-03330-1)
Supplement: Supplementary file 1 — Additional file 1: Appendix Table A.1. Results of the unconditional models; Appendix Table A.2. Average visit frequency for each category of each predictor variable in each sample; Appendix Table A.3. Models testing the options for insurance coverage and its effect on out-of-pocket visit costs; Appendix Table A.4. Full results of the final hierarchical linear models predicting chiropractic visit frequency for samples of patients with chronic low back pain and chronic neck pain. [file 12891_2020_3330_MOESM1_ESM.docx]

Additional File 1

Appendix Table A.1. Results of the unconditional models

| Estimated coefficients | Chronic low back pain – clustering variable used | | | | | Chronic neck pain – clustering variable used | | | |
| --- | --- | --- | --- | --- | --- | --- | --- | --- | --- |
|  | Region & Clinic | Region only | Clinic only | Neither | Region & Clinic | | Region only | Clinic only | Neither |
| Average number of visits per month | 2.30 (1.95, 2.64) | 2.28 (1.91, 2.66) | 2.30 (2.07, 2.53) | 2.27 (2.13, 2.42) | 2.36 (1.97, 2.76) | | 2.32 (1.95, 2.69) | 2.35 (2.11, 2.60) | 2.26 (2.11, 2.41) |
| Variance attributable to Region | 0.11 (-0.10, 0.32) | 0.18 (-0.06, 0.42) | --- | --- | 0.16 (-0.11, 0.43) | | 0.18 (-0.06, 0.42) | --- | --- |
| Variance attributable to Clinic | 0.72 (0.32, 1.13) | --- | 0.87 (0.44, 1.29) | --- | 0.84 (0.36, 1.32) | | --- | 1.05 (0.52, 1.59) | --- |
| Remaining variance | 3.93 (3.53, 4.33) | 4.53 (4.10, 4.96) | 3.92 (3.52, 4.32) | 4.72 (4.27, 5.17) | 3.16 (2.79, 3.53) | | 3.79 (3.39, 4.19) | 3.15 (2.78, 3.52) | 3.98 (3.56, 4.39) |
| Bayesian Information Criterion (BIC) | 3707 | 3737 | 3704 | 3753 | 2945 | | 2971 | 2943 | 2987 |

Appendix Table A.2. Average visit frequency for each category of each predictor variable in each sample; all values are mean (SD)

|  | Chronic Low Back Pain (n=852) | χ^2^ p-value for visit frequency difference across categories | Chronic Neck Pain  (n=705) | χ^2^ p-value for visit frequency difference across categories |
| --- | --- | --- | --- | --- |
| Mean (SD) # chiropractic visits/month | 2.3 (2.2) |  | 2.3 (2.0) |  |
| *Enabling factors* |  |  |  |  |
| Some insurance coverage for chiropractic | 2.38 (2.37) | .099 | 2.23 (1.98) | .790 |
| No insurance coverage for chiropractic | 2.01 (1.60) |  | 2.31 (2.05) |  |
| Unknown insurance coverage | 2.20 (1.82) |  | 2.45 (1.82) |  |
| *Need factors – pain characteristics* |  |  |  |  |
| Rating of pain past 7 days (0-10) <=avg of 3.66 | 1.99 (1.89) | <.001 | 1.98 (1.78) | .001 |
| Rating of pain past 7 days (0-10) >avg of 3.66 | 2.58 (2.42) |  | 2.48 (2.13) |  |
| Oswestry or Neck Disability Index score (0-100) <=avg of 20.46 | 2.02 (1.99) | <.001 | 2.04 (1.78) | .001 |
| Oswestry or Neck Disability Index score (0-100) >avg of 20.46 | 2.63 (2.36) |  | 2.54 (2.22) |  |
| Have both CLBP and CNP | 2.37 (2.14) | .155 | 2.30 (2.01) | .512 |
| Have only CLBP or CNP | 2.16 (2.21) |  | 2.20 (1.97) |  |
| Years of pain |  | .278 |  | .679 |
| Less than 1 year | 2.62 (2.32) |  | 2.49 (2.40) |  |
| 1 year to less than 2 years | 2.66 (2.24) |  | 2.60 (1.91) |  |
| 2 years to less than 5 years | 2.29 (2.51) |  | 2.26 (1.92) |  |
| 5 years to less than 10 years | 2.22 (2.21) |  | 2.12 (2.11) |  |
| 10+ years | 2.14 (2.00) |  | 2.23 (1.89) |  |
| Unknown | 2.35 (2.22) |  | 2.03 (1.62) |  |
| *Need factors – lifestyle and point in care* |  |  |  |  |
| No heavy labor | 2.07 (2.09) | .095 | 2.18 (1.88) | .617 |
| Non-workday heavy labor: >0% but <20% | 2.51 (2.64) |  | 2.18 (1.99) |  |
| Workday heavy labor: >0% but <20% | 2.59 (2.28) |  | 2.33 (2.10) |  |
| Non-workday heavy labor: >20% | 2.47 (1.49) |  | 1.93 (1.42) |  |
| Workday heavy labor: >20% | 2.10 (1.79) |  | 2.59 (2.10) |  |
| Heavy labor: missing | 2.46 (2.19) |  | 2.50 (2.53) |  |
| New patient (<30 days) | 3.14 (3.07) | <.001 | 2.67 (2.22) | .014 |
| Not a new patient | 2.09 (1.89) |  | 2.13 (1.85) |  |
| Unknown time with this chiropractor | 2.50 (2.44) |  | 2.59 (2.40) |  |
| Ended care during study period | 1.12 (1.56) | <.001 | 1.07 (1.39) | .001 |
| Did not end care during study period | 2.36 (2.19) |  | 2.31 (2.00) |  |
| *Perceived need factors – goals for treatment* |  | .048 |  | .029 |
| Goal: prevent pain getting worse | 2.28 (2.07) |  | 2.13 (2.05) |  |
| Goal: prevent pain coming back; prevent reinjury | 2.07 (2.06) |  | 2.14 (1.96) |  |
| Goal: ease or make pain go away temporarily | 2.08 (1.96) |  | 2.08 (1.75) |  |
| Goal: make pain go away permanently | 2.58 (2.45) |  | 2.62 (2.27) |  |
| Goal: other or missing | 2.62 (1.95) |  | 1.64 (0.72) |  |
| *Perceived need factors – pain beliefs* |  |  |  |  |
| Believe their pain is chronic | 2.33 (2.13) | .298 | 2.25 (2.01) | .812 |
| No, don't know, missing | 2.16 (2.27) |  | 2.29 (1.98) |  |
| What pain would be if didn’t see chiropractor 0-10 <=avg 6.77 | 1.95 (1.86) | .001 | 2.04 (1.73) | .027 |
| What pain would be if didn’t see chiropractor 0-10 >avg 6.77 | 2.47 (2.32) |  | 2.38 (2.12) |  |
| Chronic pain will never go away: agree/strongly agree | 2.40 (2.24) | .301 | 2.17 (1.96) | .448 |
| Strongly disagree, disagree, uncertain, missing | 2.23 (2.15) |  | 2.30 (2.01) |  |
| Important to understand causes of pain: agree/strongly agree | 2.28 (2.19) | .559 | 2.25 (2.00) | .569 |
| Strongly disagree, disagree, uncertain, missing | 2.10 (1.93) |  | 2.44 (1.97) |  |
| Unsafe to be physically active: agree/strongly agree | 3.31 (3.28) | <.001 | 2.60 (2.87) | .383 |
| Disagree, Strongly Disagree, uncertain, missing | 2.20 (2.06) |  | 2.25 (1.96) |  |
| *Perceived need factors – other psychological influences* |  |  |  |  |
| Pain management self-efficacy (0-10) avg<7.52 | 2.52 (2.53) | .004 | 2.34 (2.16) | .369 |
| Pain management self-efficacy (0-10) avg>7.52 | 2.08 (1.84) |  | 2.20 (1.87) |  |
| Expect chiropractic very-extremely successful | 2.27 (2.21) | .887 | 2.31 (2.03) | .271 |
| Not at all, slightly, somewhat successful, missing | 2.29 (2.08) |  | 2.11 (1.87) |  |
| Expect a lot to quite a bit of improvement | 2.39 (2.30) | .033 | 2.44 (2.08) | .002 |
| Some, a little, or no improvement, missing | 2.07 (1.92) |  | 1.95 (1.80) |  |
| Worry about pain: mod to all the time | 2.65 (2.55) | .019 | 2.63 (2.29) | .029 |
| Not at all, or to a slight degree, or missing | 2.19 (2.08) |  | 2.19 (1.93) |  |
| Has depression according to PROMIS items | 2.31 (2.17) | .793 | 2.35 (2.14) | .494 |
| Not depressed | 2.26 (2.18) |  | 2.23 (1.94) |  |
| Catastrophizing (0-12 scale) <=avg 2.31 | 2.03 (1.90) | <.001 | 2.22 (1.95) | .443 |
| Catastrophizing (0-12 scale) >avg 2.31 | 2.65 (2.49) |  | 2.33 (2.07) |  |
| *Predisposing factors* |  |  |  |  |
| Age in years <=avg 48.51 | 2.15 (2.19) | .109 | 2.28 (2.07) | .767 |
| Age in years >avg 48.51 | 2.39 (2.15) |  | 2.24 (1.91) |  |
| Age: less than 30 years | 2.34 (2.61) | .383 | 2.47 (2.22) | .099 |
| Age: 30 - 49 years | 2.11 (2.06) |  | 2.15 (1.97) |  |
| Age: 50 - 65 years | 2.37 (2.05) |  | 2.19 (1.88) |  |
| Age: 65+ years | 2.41 (2.36) |  | 2.76 (2.17) |  |
| Female | 2.16 (2.00) | .029 | 2.27 (2.02) | .807 |
| Male, other, and unknown | 2.50 (2.46) |  | 2.22 (1.89) |  |
| Education: At least a 4-year degree | 2.16 (2.07) | .108 | 2.15 (1.86) | .093 |
| Less than a 4-year degree | 2.40 (2.29) |  | 2.41 (2.17) |  |
| *Chiropractor practice characteristics* |  |  |  |  |
| Average number of patients treated per day <=avg 25.28 | 2.15 (2.01) | .029 | 2.18 (1.91) | .200 |
| Average number of patients treated per day >avg 25.28 | 2.49 (2.43) |  | 2.38 (2.12) |  |
| Percentage of patients on preventive/maintenance <=avg 23.70 | 2.18 (2.15) | .114 | 2.16 (2.03) | .096 |
| Percentage of patients on preventive/maintenance >avg 23.70 | 2.42 (2.20) |  | 2.41 (1.94) |  |
| In practice 5 to 10 years | 2.80 (2.51) | <.001 | 2.81 (2.49) | .002 |
| In practice >10 to 20 years | 2.07 (1.83) |  | 2.37 (2.05) |  |
| In practice >20 to 30 years | 1.98 (1.72) |  | 1.90 (1.51) |  |
| In practice >30 years | 2.62 (2.72) |  | 2.35 (2.15) |  |
| Clinic location is urban (>50,000 population) | 2.28 (2.23) | .844 | 2.23 (1.88) | .625 |
| Rural (<50,000 population) | 2.25 (2.08) |  | 2.31 (2.22) |  |
| Average number of physical modalities used <=avg 3.25 | 2.50 (2.32) | .004 | 2.53 (2.11) | .001 |
| Average number of physical modalities used >avg 3.25 | 2.08 (2.02) |  | 2.03 (1.87) |  |
| *College from which chiropractor graduated* |  | <.001 |  | <.001 |
| Life College | 2.95 (2.64) |  | 3.46 (2.19) |  |
| Los Angeles College of Chiropractic | 1.98 (0.98) |  | 2.71 (2.29) |  |
| National College of Chiropractic | 2.95 (3.02) |  | 2.84 (2.17) |  |
| New York Chiropractic College | 2.06 (1.70) |  | 2.02 (2.01) |  |
| Northwestern Chiropractic College | 1.97 (1.72) |  | 1.86 (1.55) |  |
| Palmer Chiropractic College | 1.96 (1.79) |  | 2.14 (1.79) |  |
| Parker Chiropractic College | 2.60 (2.45) |  | 2.87 (2.27) |  |
| Texas Chiropractic College | 2.98 (3.00) |  | 2.29 (2.17) |  |
| University of Western States | 1.73 (1.37) |  | 1.68 (1.62) |  |
| Other | 2.78 (3.13) |  | 2.95 (3.12) |  |
| *State/Region* |  | <.001 |  | <.001 |
| California | 2.81 (2.25) |  | 2.92 (2.12) |  |
| Florida | 2.40 (2.32) |  | 2.69 (2.54) |  |
| Minnesota | 1.95 (1.67) |  | 1.85 (1.55) |  |
| New York | 1.87 (1.84) |  | 2.04 (1.91) |  |
| Oregon | 1.77 (1.38) |  | 1.76 (1.70) |  |
| Texas | 2.92 (2.95) |  | 2.71 (2.27) |  |

Appendix Table A.3. Models testing the options for insurance coverage and its effect on out-of-pocket visit costs (block 1)

|  | Chronic low back pain | Chronic neck pain |
| --- | --- | --- |
| Option 1 – Coverage, Reference = no coverage |  |  |
| Some insurance coverage for chiropractic | 0.45 (0.11, 0.79) | 0.02 (-0.32, 0.36) |
| Unknown insurance coverage | 0.16 (-0.51, 0.83) | 0.05 (-0.68, 0.79) |
| Constant | 1.98 (1.65, 2.32) | 2.34 (1.99, 2.68) |
| Variance attributable to Clinic | 0.86 (0.45, 1.28) | 1.06 (0.52, 1.59) |
| Remaining variance | 3.89 (3.49, 4.28) | 3.15 (2.78, 3.51) |
| Bayesian Information Criterion (BIC) | 3710 | 2956 |
| Option 2 – Type of coverage, Reference = no coverage |  |  |
| Insurance with caps on visits or costs | 0.58 (0.20, 0.96) | 0.06 (-0.31, 0.43) |
| Insurance with no caps | 0.53 (-0.02, 1.07) | -0.17 (-0.72, 0.38) |
| Insurance with unknown caps | 0.24 (-0.17, 0.64) | 0.03 (-0.38, 0.44) |
| Unknown insurance coverage | 0.16 (-0.51, 0.82) | 0.05 (-0.69, 0.78) |
| Constant | 1.98 (1.65, 2.32) | 2.34 (2.00, 2.68) |
| Variance attributable to Clinic | 0.84 (0.42, 1.26) | 1.06 (0.52, 1.59) |
| Remaining variance | 3.88 (3.48, 4.28) | 3.14 (2.77, 3.51) |
| Bayesian Information Criterion (BIC) | 3720 | 2969 |
| Option 3 - Out-of-pocket visit costs, Reference = $1 to $20 |  |  |
| Unknown / $0 | 1.14 (0.56, 1.71) | 0.46 (-0.09, 1.00) |
| $21 to $40 | 0.33 (-0.06, 0.72) | -0.24 (-0.65, 0.16) |
| $41 to $60 | 0.23 (-0.18, 0.65) | -0.07 (-0.52, 0.38) |
| $61 to $80 | 0.08 (-0.46, 0.62) | 0.22 (-0.34, 0.78) |
| $81+ | 0.36 (-0.28, 1.01) | -0.34 (-1.06, 0.37) |
| Constant | 2.05 (1.73, 2.37) | 2.37 (2.00, 2.74) |
| Variance attributable to Clinic | 0.77 (0.38, 1.17) | 1.00 (0.48, 1.52) |
| Remaining variance | 3.87 (3.48, 4.27) | 3.12 (2.75, 3.49) |
| Bayesian Information Criterion (BIC) | 3721 | 2967 |

Appendix Table A.4. Full results of the final hierarchical linear models predicting chiropractic visit frequency for samples of patients with chronic low back pain and chronic neck pain

|  | Chronic Low Back Pain (n=852) | Chronic Neck Pain  (n=705) |
| --- | --- | --- |
| *Enabling factors - p-values for block*^1^ | *0.032* | *0.986* |
| Some insurance coverage for chiropractic – Reference = No coverage | 0.44 (0.11, 0.76) | 0.14 (-0.19, 0.46) |
| Unknown insurance coverage | 0.06 (-0.58, 0.70) | -0.15 (-0.86, 0.57) |
| *Need factors – pain characteristics - p-values for block*^1^ | *<.001* | *<.001* |
| Rating of pain past 7 days (0-10) | 0.06 (-0.04, 0.15) | 0.00 (-0.10, 0.10) |
| Oswestry or Neck Disability Index score (0-100) | 0.02 (0.00, 0.03) | 0.03 (0.01, 0.05) |
| Have both CLBP and CNP | 0.09 (-0.18, 0.37) | 0.15 (-0.13, 0.42) |
| Years of pain - Reference = <1 year |  |  |
| 1 year to less than 2 years | 0.38 (-0.24, 1.01) | 0.36 (-0.33, 1.05) |
| 2 years to less than 5 years | -0.26 (-0.79, 0.27) | -0.07 (-0.59, 0.45) |
| 5 years to less than 10 years | -0.29 (-0.80, 0.21) | -0.11 (-0.64, 0.41) |
| 10+ years | -0.39 (-0.83, 0.06) | -0.03 (-0.51, 0.44) |
| Unknown | -0.10 (-1.05, 0.86) | -0.01 (-0.94, 0.93) |
| *Need factors – lifestyle and point in care - p-values for block*^1^ | *<.001* | *<.001* |
| Heavy labor - Reference = No heavy labor |  |  |
| Non-workday heavy labor: >0% but <20% | 0.41 (-0.01, 0.84) | -0.28 (-0.74, 0.19) |
| Workday heavy labor: >0% but <20% | 0.47 (0.11, 0.84) | -0.06 (-0.41, 0.29) |
| Non-workday heavy labor: >20% | 0.23 (-0.50, 0.96) | -0.02 (-0.95, 0.91) |
| Workday heavy labor: >20% | 0.22 (-0.24, 0.69) | 0.19 (-0.31, 0.69) |
| Heavy labor: missing | 0.43 (-0.09, 0.94) | 0.30 (-0.24, 0.83) |
| New patient (<30 days) | 0.63 (0.18, 1.08) | 0.69 (0.21, 1.16) |
| Unknown time with this chiropractor | 0.32 (-0.04, 0.68) | 0.45 (0.07, 0.83) |
| Ended care during study period | -1.21 (-1.75, -0.67) | -1.57 (-2.23, -0.91) |
| *Perceived need factors – goals for treatment - p-values for block^1^* | *0.636* | *0.237* |
| Treatment goals - Reference = Preventing pain from getting worse |  |  |
| Prevent pain coming back; prevent reinjury | 0.12 (-0.34, 0.59) | 0.07 (-0.45, 0.60) |
| Ease or make pain go away temporarily | 0.02 (-0.41, 0.44) | 0.22 (-0.23, 0.67) |
| Make pain go away permanently (cure) | 0.23 (-0.21, 0.66) | 0.47 (-0.01, 0.95) |
| Other or missing | 0.64 (-0.63, 1.90) | -0.48 (-1.96, 1.01) |
| *Perceived need factors – pain beliefs - p-values for block*^1^ | *0.063* | *0.766* |
| Believe their pain is chronic | 0.11 (-0.18, 0.41) | 0.08 (-0.22, 0.38) |
| What pain would be if didn’t see chiropractor 0-10 | 0.06 (-0.01, 0.14) | 0.03 (-0.05, 0.12) |
| Chronic pain will never go away: agree/strongly agree | 0.12 (-0.18, 0.43) | 0.01 (-0.30, 0.31) |
| Important to understand causes of pain: agree/strongly agree | 0.00 (-0.55, 0.56) | -0.41 (-1.01, 0.20) |
| Unsafe to be physically active: agree/strongly agree | 0.56 (0.01, 1.11) | -0.19 (-0.93, 0.55) |
| *Perceived need factors – other psychological factors - p-values for block*^1^ | *0.295* | *0.100* |
| Pain management self-efficacy (0-10) | -0.04 (-0.12, 0.05) | 0.05 (-0.04, 0.15) |
| Expect chiropractic very-extremely successful | 0.14 (-0.20, 0.48) | 0.03 (-0.33, 0.39) |
| Expect a lot to quite a bit of improvement | 0.18 (-0.13, 0.49) | 0.32 (0.01, 0.63) |
| Worry about pain: mod to all the time | -0.05 (-0.53, 0.42) | 0.31 (-0.19, 0.82) |
| Has depression according to PROMIS items | -0.30 (-0.62, 0.02) | -0.15 (-0.46, 0.17) |
| Catastrophizing (0-12 scale) | 0.01 (-0.08, 0.10) | -0.06 (-0.15, 0.03) |
| *Predisposing factors - p-values for block*^1^ | *0.048* | *0.171* |
| Age in years | 0.01 (-0.00, 0.02) | 0.01 (-0.00, 0.02) |
| Female | -0.37 (-0.65, -0.08) | -0.12 (-0.47, 0.24) |
| Education: At least a 4-year degree | 0.04 (-0.25, 0.32) | -0.15 (-0.43, 0.13) |
| *Chiropractor practice characteristics - p-values for block*^1^ | *<.001* | *<.001* |
| Average number of patients treated per day | 0.03 (0.01, 0.04) | 0.04 (0.02, 0.06) |
| Percentage of patients on preventive/maintenance | 0.01 (-0.00, 0.02) | 0.01 (0.00, 0.02) |
| Years in practice - Reference = 5 to 10 years |  |  |
| In practice >10 to 20 years | -0.50 (-1.05, 0.05) | -0.16 (-0.72, 0.39) |
| In practice >20 to 30 years | -0.72 (-1.30, -0.15) | -1.01 (-1.61, -0.41) |
| In practice >30 years | 0.24 (-0.43, 0.91) | -0.13 (-0.83, 0.56) |
| Clinic location is urban (>50,000 population) | -0.20 (-0.59, 0.19) | -0.18 (-0.60, 0.23) |
| Average number of physical modalities used | -0.12 (-0.26, 0.01) | -0.04 (-0.18, 0.11) |
| *College from which chiropractor graduated - p-value for block*^1^ | *0.130* | *0.060* |
| Reference = Life College |  |  |
| Los Angeles College of Chiropractic | -1.34 (-2.36, -0.32) | -0.21 (-1.22, 0.80) |
| National College of Chiropractic | 0.06 (-0.89, 1.01) | -0.29 (-1.26, 0.68) |
| New York Chiropractic College | 0.38 (-0.63, 1.39) | 0.13 (-1.02, 1.28) |
| Northwestern Chiropractic College | -0.40 (-2.73, 1.93) | 0.02 (-2.07, 2.11) |
| Palmer Chiropractic College | -0.89 (-1.71, -0.06) | -0.75 (-1.60, 0.11) |
| Parker Chiropractic College | -0.20 (-1.35, 0.96) | -0.48 (-1.70, 0.73) |
| Texas Chiropractic College | -0.45 (-1.59, 0.69) | -0.76 (-1.97, 0.44) |
| University of Western States | -1.45 (-3.22, 0.33) | -1.46 (-3.19, 0.27) |
| Other | 0.41 (-0.60, 1.42) | 0.41 (-0.66, 1.47) |
| *State/Region - p-value for block*^1^ | *0.014* | *0.072* |
| State/Region - Reference = California |  |  |
| Florida | -0.67 (-1.46, 0.12) | -0.66 (-1.47, 0.15) |
| Minnesota | -0.57 (-2.88, 1.73) | -1.31 (-3.33, 0.72) |
| New York | -1.49 (-2.34, -0.65) | -1.20 (-2.11, -0.28) |
| Oregon | 0.48 (-1.13, 2.09) | 0.20 (-1.34, 1.73) |
| Texas | -0.30 (-1.34, 0.74) | -0.05 (-1.10, 1.00) |
| Constant | 1.45 (-0.11, 3.01) | 0.70 (-0.98, 2.37) |
| Remaining variance attributable to chiropractor/clinic | 0.19 (-0.00, 0.37) | 0.23 (-0.00, 0.47) |
| Remaining variance not attributable to chiropractor/clinic | 3.45 (3.10, 3.80) | 2.79 (2.46, 3.11) |

^1^P-value for the likelihood-ratio test of the incremental explanatory power of each block of variables added to a model containing all previous blocks.
